# Some *ABCA3 *mutations elevate ER stress and initiate apoptosis of lung epithelial cells

**DOI:** 10.1186/1465-9921-12-4

**Published:** 2011-01-07

**Authors:** Nina Weichert, Eva Kaltenborn, Andreas Hector, Markus Woischnik, Andrea Schams, Andreas Holzinger, Sunčana Kern, Matthias Griese

**Affiliations:** 1 Pediatric Pneumology, Dr. von Hauner Children's Hospital, Ludwig-Maximilians University, Munich, Germany; 2 Neonatology, Dr. von Hauner Children's Hospital, Ludwig-Maximilians University, Munich, Germany

## Abstract

**Background:**

ABCA3 transporter (ATP-binding cassette transporter of the A subfamily) is localized to the limiting membrane of lamellar bodies, organelles for assembly and storage of pulmonary surfactant in alveolar epithelial type II cells (AECII). It transports surfactant phospholipids into lamellar bodies and absence of ABCA3 function disrupts lamellar body biogenesis. Mutations of the *ABCA3 *gene lead to fatal neonatal surfactant deficiency and chronic interstitial lung disease (ILD) of children. *ABCA3 *mutations can result in either functional defects of the correctly localized ABCA3 or trafficking/folding defects where mutated ABCA3 remains in the endoplasmic reticulum (ER).

**Methods:**

Human alveolar epithelial A549 cells were transfected with vectors expressing wild-type ABCA3 or one of the three ABCA3 mutant forms, R43L, R280C and L101P, C-terminally tagged with YFP or hemagglutinin-tag. Localization/trafficking properties were analyzed by immunofluorescence and ABCA3 deglycosylation. Uptake of fluorescent NBD-labeled lipids into lamellar bodies was used as a functional assay. ER stress and apoptotic signaling were examined through RT-PCR based analyses of XBP1 splicing, immunoblotting or FACS analyses of stress/apoptosis proteins, Annexin V surface staining and determination of the intracellular glutathion level.

**Results:**

We demonstrate that two *ABCA3 *mutations, which affect ABCA3 protein trafficking/folding and lead to partial (R280C) or complete (L101P) retention of ABCA3 in the ER compartment, can elevate ER stress and susceptibility to it and induce apoptotic markers in the cultured lung epithelial A549 cells. R43L mutation, resulting in a functional defect of the properly localized ABCA3, had no effect on intracellular stress and apoptotic signaling.

**Conclusion:**

Our data suggest that expression of partially or completely ER localized ABCA3 mutant proteins can increase the apoptotic cell death of the affected cells, which are factors that might contribute to the pathogenesis of genetic ILD.

## Background

ABCA3 is a member of the ATP-binding cassette (ABC) family of transporters which utilize the energy of ATP hydrolyses to drive the transport of a variety of substrates across biological membranes [1]. The *ABCA3 *gene is highly expressed in alveolar epithelial type II cells (AECII) of the lung [2,3]. In AECII ABCA3 protein localizes to the limiting membrane of lamellar bodies as lipid-rich organelles for production, storage and secretion of pulmonary surfactant [4,5]. Surfactant is a complex mixture of 90% lipids (mostly phospholipids) and 10% surfactant-specific proteins (e.g. small hydrophobic proteins SP-B and SP-C) produced by AECII which reduces surface tension on the air-liquid interface and prevents alveolar collapse at the end of expiration. ABCA3 is a lipid transporter which transports surfactant phospholipids into lamellar bodies where surfactant is assembled. It is essential for lamellar body biogenesis [6-9] so patients with *ABCA3 *mutations and *Abca3 *knock-out mouse have distinctive dense inclusions within immature lamellar bodies and disturbed composition of surfactant phospholipids [7,8,10-13]. Since the last steps in SP-B and SP-C processing occur inside of functional lamellar bodies, ABCA3 deficiency in human and mouse leads to accumulation of SP-B and SP-C precursors [7,8,14,15].

In 2004 mutations of the *ABCA3 *gene were recognized as a cause of lung diseases in full-term neonates with fatal pulmonary surfactant deficiency [10]. Today *ABCA3 *mutations are known to cause also chronic interstitial lung disease (ILD) in children and older patients [12,16,17]. With more than 100 identified mutations, ABCA3 is the most frequent known cause of genetic ILD (own unpublished data), [12,16,17]. Similar to SP-C deficiency, ABCA3-related ILD is complex and heterogeneous in histopathology and symptom severity. The disease onset varies from directly after birth, early in infancy or later in childhood [10,12,16,18,19], sometimes following the exposure to environmental stressors such as cigarette smoke [12,16].

*ABCA3 *mutations classify either as functional defects of properly localized proteins or trafficking/folding defects where misfolded proteins accumulate in the ER [6,20]. Folding of newly synthesized proteins is a highly controlled process happening in the ER lumen with assistance of molecular chaperones. Proteins which fail to fold properly are harmful for the cell and retained inside the ER by the ER quality control. ER accumulation of misfolded proteins causes ER stress and activates cytoprotective mechanisms named unfolded protein response (UPR). UPR promotes the ER protein folding capacity by increasing the production of molecular chaperones and attenuates general protein translation to decrease the misfolded protein load in the ER [21]. If UPR fails to resolve ER stress and restore cell homeostasis, the cell will be eliminated by initiation of tightly controlled apoptotic cell-death pathways [22].

Recent data show that ER stress and apoptosis of AECII play a role in lung disease, especially in pathogenesis of idiopathic pulmonary fibrosis (IPF) and genetic SP-C-associated pulmonary fibrosis [23-26]. Moreover, cultured lung epithelial A549 cells expressing SP-C mutations, which cause misfolding and aggregation of the SP-C pre-protein, increase ER stress, activate UPR and initiate apoptotic cell-death [26-28]. Fibrosis is one of the hallmarks documented in ABCA3-associated ILD [12,16,17] and knowing that *ABCA3 *mutations can cause ER retention of the mutated transporter [6,20], we investigated the influence of three *ABCA3 *mutations, R43L, R280C and L101P, found in children with surfactant deficiency and chronic ILD [10,14,19], on ER stress and apoptosis induction in lung epithelial A549 cells.

## Methods

### Plasmid Vectors

Wild type (WT) full length human *hABCA3 *cDNA without stop codon was cloned into *EcoR*I/*Age*I sites of pEYFP-N1 plasmid (Clontech, Mountain View, CA) to obtain pEYFP-N1/WT vector for expression of C-terminal ABCA3-YFP protein fusions. pUB6-HA/WT vector for expression of C-terminal fusions of ABCA3 with hemagglutinin tag (HA-tag) was produced by modification of pUB6/V5-His vector (Invitrogen, Karlsruhe, Germany). His-tag was put out of frame and WT h*ABCA3 *cDNA with 3' HA-tag sequence (5'-TAC CCA TAC GAT GTT CCA GAT TAC GCT-3') was cloned into *Kpn*I/*Xho*I restriction sites. Three *hABCA3 *point mutations R43L, R280C and L101P were introduced in the WT *ABCA3 *in both vector types by PCR-based site-directed mutagenesis (QuickChange Site-Directed Mutagenesis, Stratagene, La Jolla, CA). Mutagenesis primers were as follows: *R43L-For *5'-CAT CTG GCT CC**T**CTT GAA GAT TC-3', *R43L-Rev *5'-GAA TCT TCA AG**A**GGA GCC AGA TG-3', *L101P-For *5'-CAG TGC GCA GGG CAC **C**TG TGA TCA AC-3', *L101P-Rev *5'-GTT GAT CAC A**G**G TGC CCT GCG CAC TG-3', *R280C-For *5'-CAT TGC C**T**G TGC TGT CGT G-3', *R280C-Rev *5'-CAC GAC AGC AC**A**GGC AAT G-3'. Successful mutagenesis in pEYFP-N1/ABCA3 and pUB6-HA/ABCA3 (ABCA3 denotes WT or one of the three mutations) was confirmed by sequencing.

### Cell Culture and Transfection

Human lung carcinoma epithelial cell line A549 (ACC 107) was obtained from the German Collection of Microorganisms and Cell Cultures (DSMZ, Braunschweig, Germany). Cells were grown in RPMI 1640 medium with 10% FBS. At 70% confluence cells were transiently transfected with pEYFP-N1/ABCA3 or pUB6-HA/ABCA3 vectors using ExGen 500 (Fermentas, Burlington, ON) according to the manufacturer's protocol. 48 h after transfection samples were collected for experiments. Transfection efficiency was confirmed in all samples through YFP signal or immunofluorescence of HA-tag (see bellow). ABCA3 construct expression was similar in all transfected cells as assessed by ABCA3 mRNA and protein expression levels. ABCA3 mRNA expression after transfection strongly increased (>30-fold) compared to non-transfected A549 cells (data not shown). When indicated transfected cells were incubated 14 h with 10 μg/ml of tunicamycin (Sigma, St. Louis, MO) or 16 h with 25 ng/ml of TNFα (Invitrogen) prior to sample collection.

### Immunofluorescence

Cells grown on coverslips were transfected with pEYFP-N1/ABCA3 or pUB6-HA/ABCA3 vectors, washed in PBS buffer, fixed in 4% paraformaldehyde, permeabilised and incubated 1 h at room temperature with the following primary antibodies: monoclonal mouse anti-LAMP3/CD63 (1:200, Chemicon, Tamecula, CA), polyclonal goat anti-calnexin (1:200, Santa Cruz Biotechnology, Santa Cruz, CA) and to detect HA-tagged ABCA3 monoclonal rat anti-HA-tag (1:200, Roche, Manheim, Germany). Signals were visualized with Alexa Fluor 555 anti-goat or Alexa Fluor 555 anti-mouse IgG secondary antibodies (1:200, Invitrogen). Alexa Fluor 555 and YFP fluorescence of mounted samples was examined with Axiovert 135 fluorescent microscope and evaluated with AxioVision 4.7.1 software (Carl Zeiss, Jena, Germany).

### Immunoblotting

Cells were collected and resuspended in lysis buffer (0.15 M NaCl, 1% Triton-X100, 0.5% sodium deoxycholate, 50 mM Tris, 5 mM EDTA) supplied with protease inhibitor cocktail (Complete; Roche). 30 μg of total protein was transferred to a PVDF membrane and immunoblotted with the following primary antibodies: monoclonal mouse anti-GFP (1:500, Clontech), monoclonal rabbit anti-BiP/Grp78 (1:1000, Cell Signaling, Frankfurt, Germany), monoclonal mouse anti-caspase 4 (1:500, Stressgene), and monoclonal mouse anti-β-actin HRP conjugate (1:10000, Santa Cruz). Signal was detected using chemiluminiscent labeling with Amersham ECL Detection Reagents (GE Healthcare, Buckinghamshire, UK).

### Membrane Isolation and Deglycosylation Assay

For crude membrane preparation cells transfected with pEYFP-N1/ABCA3 vectors were collected in PBS supplemented with 1 mM EDTA and protease inhibitor (Complete, Roche). Cells were broken in a Potter-Elvehjem homogenizer and subsequently sonicated (Branson Digital Sonifier S450D) while keeping the samples on ice. Samples were centrifuged at 1000 × g, 10 min, 4 °C and obtained postnuclear supernatants were centrifuged at 100 000 × g, 1 h, 4 °C. Membrane pellets were resuspended in 25 mM Hepes/NaOH, pH 7.0 with protease inhibitor and stored at -80°C. For the deglycosylation assay 5 μg of membranes were incubated 1 h at 37 °C with PNGaseF or EndoH (New England Biolabs, Ipswich, MA). The samples were separated on 3-8% SDS-PAGE gels (NuPAGE; Invitrogen) and immunoblotted with monoclonal mouse anti-GFP antibody (1:500, Clontech).

### Liposome Preparation and NBD-Lipid Uptake

NBD-labeled lipids phosphatidylcholine (C_12_-NBD-PC) and phosphatidylethanolamine (C_12_-NBD-PE) were purchased from Avanti Polar Lipids (Alabaster, AL), other lipids from Sigma. Liposome preparation and NBD-lipid uptake were performed following previously published protocols [6]. Liposomes (~100 nm) containing C_12_-NBD-PC or C_12_-NBD-PE were prepared by mixing lipids in chloroform in the following molar ratios: L-αDPPC: C_12_-NBD-PC: egg PC: egg PG: cholesterol = 5:5:5:3:2 and L-αDPPC: egg PC: egg PG: cholesterol: C_12_-NBD-PE = 10:5:3:2:2. Cells grown on coverslips were transfected with pUB6-HA vectors expressing ABCA3 WT or one of the three mutations. 48 h later cells were incubated for 2 h with liposomes so that the final concentration of C_12_-NBD-PC or C_12_-NBD-PE in the medium was 150 μM. Cells were washed three times with PBS and prepared for immunofluorescence with primary monoclonal rat anti-HA-tag antibody (1:200, Roche) and secondary anti-rat IgG Alexa Fluor 555 antibody (1:200, Invitrogen). NBD-lipid uptake was examined with an Olympus FluoView FV 1000 confocal microscope.

### RT-PCR and XBP1 Splicing

A549 cells were transfected with YFP vectors and where indicated treated 14 h with 10 μg/ml of tunicamycin (Sigma). 48 h after transfection total RNA was isolated (High Pure RNA Isolation Kit, Roche) and cDNA was synthesized (SuperScript III First-Strand Synthesis System, Invitrogen). For every RNA sample a control reaction without reverse transcriptase was performed to exclude genomic DNA contamination. cDNA was further used as a template for PCR with XBP1 and 18S rRNA primers as published in [29]. For easier evaluation half of the PCR product was digested with *Pst*I endonuclease cutting only inside of the 26 nt intron removed from the *XBP1 *mRNA by splicing. Cut and uncut PCR products were analyzed on a 3% agarose gel. Results were calculated as the ratio of the spliced (s) and unspliced (u) band (s/u; without *Pst*I digestion) and additionally confirmed by calculating the ratio of the spliced band and the sum of the two *Pst*I digest bands (s/(u1+u2), with *Pst*I digestion). Hybrid band (h) was considered as equally contributing to unspliced and spliced bands.

### FACS Analyses

Cells transfected with pEYFP-N1/ABCA3 vectors were gated in the FL-1 channel (YFP-positive population of cells) and apoptosis was determined using different approaches. Early apoptosis was assayed by 1) measuring intracellular glutathione (GSH) levels with the cell-permeable monochlorobimane (Sigma) method [30] and 2) by annexin V^+^/propidium iodide (PI)^- ^staining (Cy5-conjugated anti-annexin V and PI; BD Bioscience, Heidelberg, Germany), and late apoptosis was determined via intracellular active caspase 3 levels (PE-conjugated anti-active-caspase 3; BD Bioscience) in cells permeabilized with the IntraPrep kit (Beckman Coulter, Krefeld, Germany) according to the manufacturer's protocol. To exclude unspecific binding isotype controls for caspase 3 and negative controls for glutathione and annexin V/PI staining were applied. BD FACSCanto II Flow Cytometer was used for the assay and FACSDiva v6.1.3 for data analyses (BD Bioscience).

### Statistical Analyses

Statistical analysis was performed by one-way ANOVA and Bonferroni's test using GraphPad Prism version 4.0 (GrapPad Software Inc., San Diego, CA). All results were presented as means ± SEM of minimum four experiments and p-values < 0.05 were considered significant.

## Results

### General characterization of R43L, R280C and L101P ABCA3 mutations

#### A) Localization and trafficking

Three clinically relevant *ABCA3 *mutations identified in patients with neonatal surfactant deficiency (R43L and L101P) and chronic ILD (R280C) were chosen for the study. While cell biology of R43L and R280C mutations has not been studied yet, L101P mutation was previously described as a trafficking/folding defect resulting in the ER accumulation of L101P protein [6,20] and was deliberately chosen for this study as a cause for the ABCA3 ER retention.

**Figure 1:**
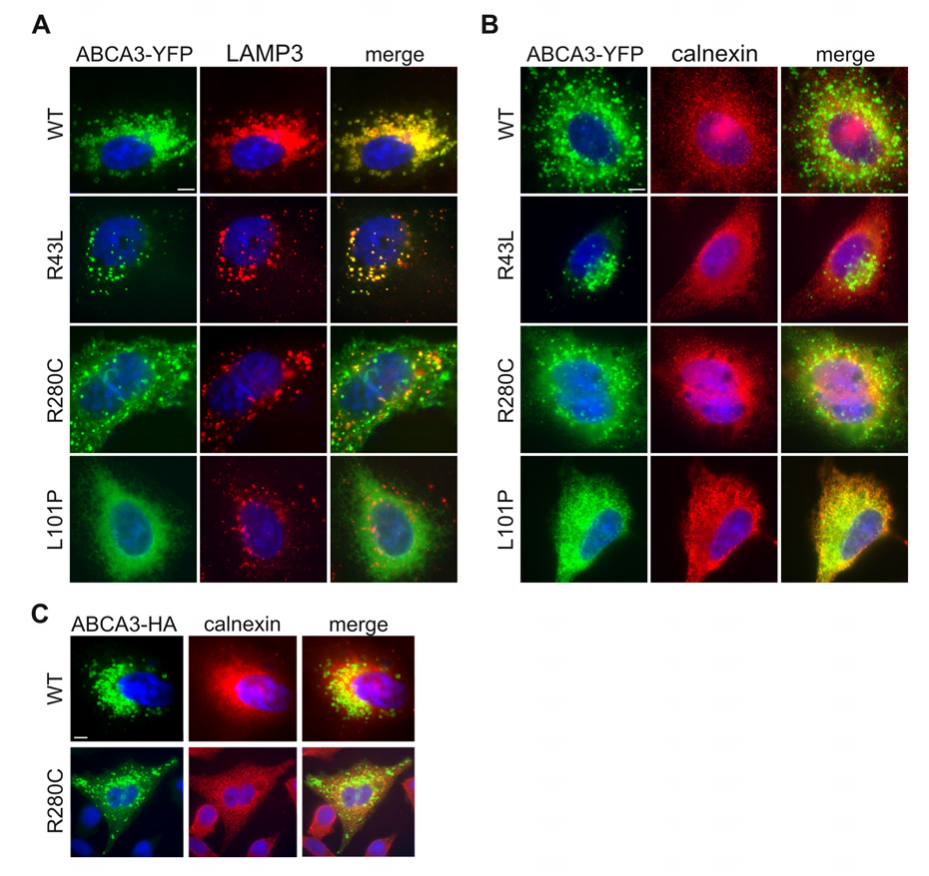
**Intracellular localization of the WT and mutant R43L, R280C and L101P ABCA3 proteins**. A549 cells were transiently transfected with pEYFP-N1/ABCA3 vectors expressing WT or one of the three mutations. YFP fluorescence of ABCA3-YFP fusions (*green*) was used to detect ABCA3 WT and R43L, R280C and L101P proteins. Colocalization of the ABCA3-YFP signal with the fluorescent signal of (A) the lysosomal/lamellar body protein LAMP3 and (B) the ER protein calnexin (*both red*) are shown. WT and R43L localized in LAMP3^+ ^vesicles, R280C partially in LAMP3^+ ^vesicles and partially in the ER and L101P completely in the ER. (C) Partial ER localization of HA-tagged R280C in A549 cells transfected with pUB6/HA-R280C plasmid confirms the partial R280C ER retention as independent on the type of the plasmid or protein tag. Representative pictures are shown; scale bars: 5 μm.

Initially we investigated intracellular localization of the WT and R43L, R280C and L101P transporters. We transfected A549 cells with two types of vectors expressing YFP (pEYFP-N1/ABCA3) or HA tagged ABCA3 (pUB6-HA/ABCA3). Fluorescence microscopy of A549 cells expressing either type of protein fusions showed no differences in the protein behavior depending on the type of the vector or the size of the protein tag (Figure 1A, B and 1C). WT protein colocalized with lysosomal-associated membrane protein 3 (LAMP3), a marker for lamellar bodies and lamellar-body-like vesicles (Figure 1A) [5,6,20]. Similar was observed for the R43L mutant, which showed a vesicular signal that overlapped with LAMP3 fluorescence (Figure 1A). For both WT and R43L mutant, very little to no colocalization was detected with calnexin (Figure 1B). WT and R43L might colocalize with the ER-resident protein calnexin during their folding in the ER as expected during the protein maturation process. R280C protein colocalized frequently with LAMP3, however the colocalization was not absolute, showing often cytoplasmic distribution that overlapped with the fluorescence of calnexin (Figure 1A, B). L101P mutation did not show any vesicular signal at all (Figure 1A) but presented with a cytoplasmic fluorescence mainly colocalizing with the ER protein calnexin (Figure 1B). This suggests correct localization of the WT and R43L transporters in the LAMP3-positive (LAMP3^+^) vesicles and almost full retention of the L101P mutant in the ER, possibly as a result of protein misfolding. Dual localization of R280C protein might be a sign of hindrance in the processing and folding of this mutant which slows down but does not abolish its progress through the ER, Golgi and toward LAMP3^+ ^vesicles.

#### B) Processing and maturation

**Figure 2:**
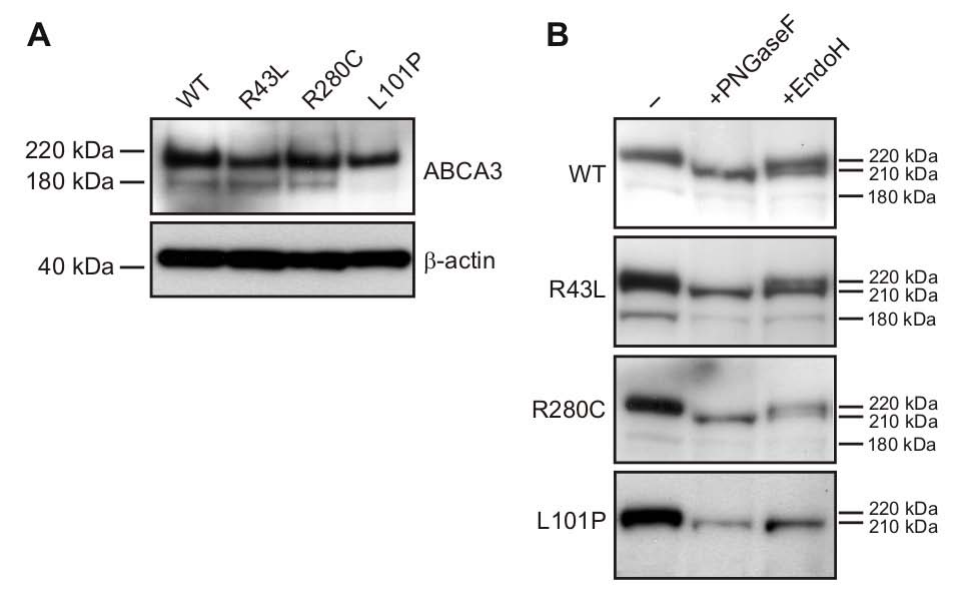
**Processing of the WT and mutant R43L, R280C and L101P ABCA3**. (A) Immunodetection with anti-GFP antibody showed two ABCA3 protein bands (180 kDa and 220 kDa) in whole cell lysates of A549 cells expressing WT and R43L and R280C mutations, and only one protein band in the cells expressing L101P mutation. (B) Deglycosylation assay with PNGaseF and EndoH on the membrane fractions from A549 cells transfected with pEYFP-N1/ABCA3 plasmids and subsequent ABCA3-YFP immunodetection with anti-GFP antibody showed presence of high-mannose and complex oligosaccharides in WT, R43L and R280C proteins, as well as only high-mannose and no complex oligosaccharides in the L101P mutant resulting from the L101P ER retention.

In immunoblots with anti-GFP antibody on cell lysates from transfected A549 cells expressing YFP labeled WT, R43L and R280C proteins, two protein bands of 180 kDa (150 kDa ABCA3 plus 30 kDa YFP) and 220 kDa (190 kDa ABCA3 plus 30 kDa YFP) were detected (Figure 2A). They might represent ABCA3 processing forms, as previously suggested, with the active ABCA3 form still unknown [6,20,29]. The processing of ER retained L101P protein was different, showing complete lack of the 180 kDa band, in line with published data (Figure 2A) [6,20].

ABCA3 is a glycoprotein that obtains high-mannose oligosaccharides inside the ER and complex oligosaccharides by modification of high-mannose oligosaccharides in the Golgi. Processing of oligosaccharides and protein progress down the ER-Golgi maturation pathway was analyzed by deglycosylation of membrane fractions from A549 cells expressing ABCA3 WT and three mutations with two endoglycosidases EndoH (cleaves only high-mannose sugars) and PNGaseF (cleaves high-mannose and complex sugars) (Figure 2B). Immunoblotting with anti-GFP antibody revealed absence of complex sugars in L101P protein that was susceptible to both enzymes, PNGaseF and EndoH, resulting in both cases in a single shifted deglycosylated 210 kDa band and no 220 kDa band. Both sugar types were present in WT, R43L and R280C proteins, as visible by the resistance of a portion of the 220 kDa band to the EndoH treatment. The 180 kDa band was resistant to deglycosylation (Figure 2B). This confirms the localization studies showing retention of the L101P mutant in the ER and ability of WT, R43L and R280C to progress further from the ER to the Golgi.

#### C) Functional assay

**Figure 3:**
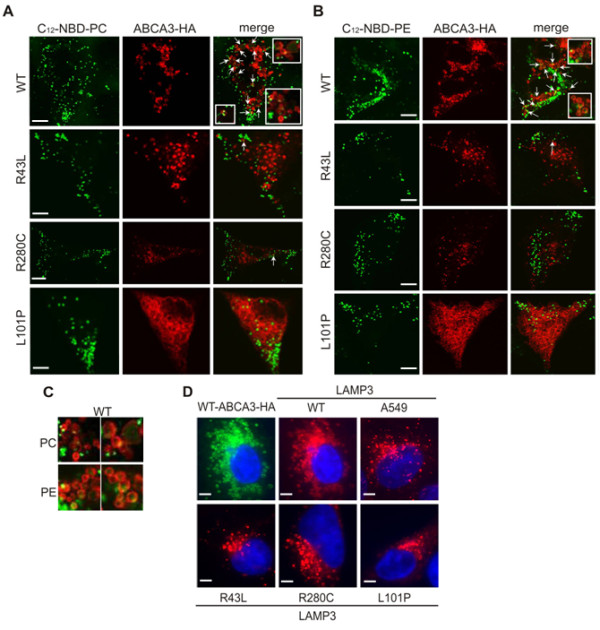
**Function of the R43L and R280C transporters**. (A-C) Uptake of C_12_-NBD-phospholipids PC and PE into ABCA3 vesicles in A549 cells transfected with pUB6/HA-ABCA3 vectors was studied by confocal microscopy. Transfected cells were incubated for 2 h with (A) C_12_-NBD-PC and (B) C_12_-NBD-PE containing liposomes (150 μM of NBD-lipid). HA-tag was used to detect ABCA3 WT and R43L, R280C and L101P by immunofluorescence (*red*). Both C_12_-NBD-PC and C_12_-NBD-PE fluorescence (*green*) frequently colocalized with the ring-like ABCA3 WT signal as well as within the ABCA3-WT vesicles and almost never with the R43L and R280C vesicles. In small sections and in (C) uptake into ABCA3-WT vesicles of both NBD-lipids PC and PE is depicted closer. Uptake through the plasma membrane into the cytoplasm of L101P transfected cells with no functional exogenous ABCA3 was observed as well as numerous cytoplasmic NBD-positive liposome-like vesicles in all A549 cells independent of the ABCA3 mutation shown in (A) and (B). (D) Influence of WT ABCA3 and three mutations on biogenesis of LAMP3^+ ^vesicles in A549 transfected with pUB6/HA-ABCA3. WT ABCA3 (*green*) induced biogenesis of LAMP3^+ ^vesicles (*red*) increasing their number and size in A549 cells, while R43L, R280C and L101P proteins showed no such effect (the same was observed in A549 pEYFP-N1/ABCA3 transfected cells - not shown). Representative pictures are shown; scale bars: 5 μm.

Trafficking/folding defect and ER accumulation of L101P protein exclude the ABCA3 function in the case of this mutant. However, R43L and R280C mutation are mostly (R280C) or completely (R43L) correctly localized and potentially functional (Figure 1A, B). Therefore, we undertook a functional study of uptake of fluorescently NBD-labeled lipids into the lamellar-body-like vesicles in A549 cells transfected with pUB6-HA/ABCA3 plasmids (Figure 3). Liposomes containing NBD-labeled major surfactant phospholipid phosphatidyl-choline (C_12_-NBD-PC) and NBD-labeled minor surfactant phospholipid phosphatidylethanol-amine (C_12_-NBD-PE) were incubated with A549 cells expressing WT, R43L, R280C and L101P HA-tagged proteins. L101P mutant was used to monitor the situation with nonfunctional ABCA3 protein. Colocalization of the fluorescent signal of C_12_-NBD-PC or C_12_-NBD-PE (green) with ABCA3-HA-positive vesicles (red), which normally colocalized with LAMP3 (Figure 1A), was monitored by confocal microscopy.

Interestingly, uptake of fluorescent liposomes into A549 cells was prominent in all cells, including those expressing L101P mutants (Figure 3) and therefore without ABCA3 function and untransfected A549 (data not shown), showing numerous NBD-positive vesicular structures dispersed throughout the cytoplasm (Figure 3). This confirms that lipid/liposome uptake into the cytoplasm through the plasma membrane does not depend on functional ABCA3, and that ABCA3 is solely an intracellular vesicular lipid transporter.

WT-ABCA3-HA signal formed ring-like structures, consistent with the localization of ABCA3 in the limiting membrane of lamellar bodies. Both NBD-PC and NBD-PE fluorescent signals were often visible as a punctual signal merging with this ring-like border of WT-ABCA3-HA vesicles (Figure 3A, B and 3C). Also, accumulation of the weaker diffuse green NBD signal was frequently observed within the inner space of WT-ABCA3-HA vesicles (Figure 3C and small sections in Figure 3A, B). Similar colocalization of NBD fluorescence with ABCA3-HA vesicles was extremely rarely observed in cells expressing R43L and R280C mutations (Figure 3A, B). This probably indicates the ability of WT-ABCA3-HA vesicles to take up and accumulate both fluorescent lipids, while R280C-ABCA3-HA and R43L-ABCA3-HA vesicles did not show such ability.

#### D) Biogenesis of LAMP3^+ ^vesicles

Expression of WT or three mutations exhibited different effects on biogenesis of the LAMP3^+ ^vesicles in A549 cells. In A549 cells, that normally show a low number of small LAMP3^+ ^vesicles, expression of WT-ABCA3 significantly induced biogenesis of numerous big vesicles with ring-like signals of ABCA3 fluorescence (equally from pEYFP-N1 or pUB6-HA vectors). Expression of R43L, R280C and L101P mutations had a negative effect on vesicle formation and induced a lower number of smaller compact LAMP3^+ ^vesicles, with the most drastic effect in L101P mutant (Figure 3D). This again proves the role of ABCA3 in lamellar body biogenesis [6,7,29]. Decreased uptake of NBD fluorescence in lamellar bodies and impact on lamellar body biogenesis together suggest functional impairment of the R43L and R280C proteins.

### L101P and R280C mutation upregulate ER stress marker BiP

**Figure 4:**
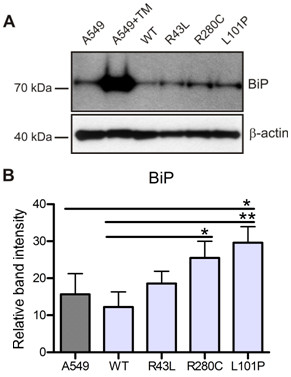
**Increase in the ER stress chaperone BiP in A549 cells expressing R280C and L101P mutations**. (A) A549 cells with ER retained L101P mutant or partially ER retained R280C protein showed upregulation of the immunodetected ER chaperone BiP in comparison to WT and A549 cells. BiP was drastically upregulated after 14 h treatment of A549 cells with 10 μg/ml of the ER stressor tunicamycin (TM). (B) Densitometric quantification of the BiP protein level of four independent experiments, without the drastic effect of TM treatment, is presented; * p < 0.05, ** p < 0.01.

BiP/Grp78 is an essential ER chaperone of the Hsp70 family, which assists the translocation of a nascent protein chain into the ER and its subsequent folding. When misfolded proteins accumulate in the ER, BiP upregulation is one of the first signals of ER stress and allows further UPR activation [30]. Immunoblotting of whole cell lysates from A549 cells expressing ABCA3-WT and three mutants revealed significant upregulation of BiP chaperone in the case of L101P mutation and R280C mutation in comparison to WT, caused by complete (L101P) or partial ER retention (R280C) of these two mutated ABCA3 transporters (Figure 4A, B). No significant BiP increase was detectable between WT and correctly localized R43L mutation. While treatment of A549 cells with a potent ER stress-causing antibiotic tunicamycin (TM) caused drastic BiP upregulation in A549 cells, differences measured between WT and three mutations were more subtle.

### L101P and R280C mutations increase susceptibility of A549 cells to ER stress

**Figure 5:**
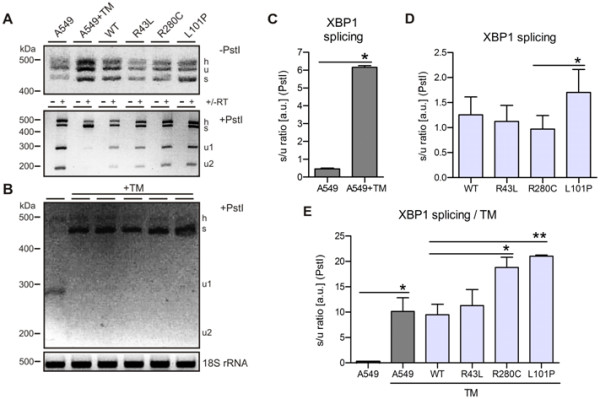
**Susceptibility of A549 cells with *ABCA3 *mutations to ER stress measured by XBP1 splicing**. XBP1 activation during UPR is regulated on the level of splicing of a 26 nt intron from the *XBP1 *mRNA. (A) XBP1 splicing in untransfected A549 cells with and without tunicamycin (TM) treatment (10 μg/ml, 14 h) and in A549 cells with R43L, R280C and L101P mutations. Two PCR products were detected by RT-PCR: **u **- unspliced and **s **- spliced XBP1 bands. **h **denotes a hybrid band between unspliced and spliced ssDNA produced during PCR and observed after long-run separation on 3% agarose (*upper panel*) (21). For easier densitometric evaluation and to ensure good separation between unspliced and hybrid band, half of the PCR products were cut by *Pst*I endonuclease which cuts only unspliced (u) band giving two bands **u1 **and **u2 **(*middle panel*). Control reactions without reverse transcriptase (-RT) are shown as well. (C, D) Densitometric quantification of the bands (s, u) in (A) from the middle panel (*Pst*I digest), presented as the ratio s/(u1+u2). TM highly elevated XBP1 splicing in A549 cells. Lower effect of L101P mutation on XBP1 splicing in A549 cells and no effect in A549 with WT and R43L and R280C mutations were observed. (B, E) TM treatment (10 μg/ml, 14 h) strongly induced XBP1 splicing (disappearance of unspliced bands u1 and u2) in all A549 cells expressing ABCA3 mutations, with the most significant increase in A549 expressing R280C and L101P mutations (increase in spliced band s). Shown are (B) 3% agarose gel after *Pst*I digestion of the RT-PCR products (*upper panel*) and 18S rRNA (*lower panel*), and (E) densitometric evaluation of the bands. Presented graphs are the densitometric quantification of four independent experiments; * p < 0.05, ** p < 0.01.

Upon ER accumulation of misfolded proteins BiP dissociates from the luminal domain of IRE1, allows IRE1 dimerization and synthesis of active XBP1 protein, a UPR transcription factor which regulates expression of ER stress proteins including BiP. XBP1 activation is regulated on the level of splicing of a 26 nt intron from the *XBP1 *mRNA by endoribonuclease activity of the IRE1 dimer [31]. To confirm previous observation on BiP upregulation and presence of intracellular stress, we assayed splicing of the *XBP1 *mRNA by RT-PCR [32]. We have measured the ratio of spliced (s; ~500 bp - 26 bp) and unspliced (u; ~500 bp) XBP1 RT-PCR bands, prior and after the treatment of the PCR products with *Pst*I restriction endonuclease, as a measuring control. A slower migrating hybrid (h) band of unspliced and spliced single-stranded DNAs produced during PCR, and thus equally contributing to unspliced and spliced bands, was observed as well after the long-run separation on 3% agarose (Figure 5A) [33]. Tunicamycin treatment strongly increased the intensity of the spliced PCR band in A549 cells in comparison to untreated A549 (Figure 5C). In A549 cells with either WT or one of the three mutations, increase of XBP1 splicing was measured only in the case of L101P mutation (Figure 5D). Probably because of the robustness of the method, finer differences between WT and R43L and R280C were not observable, and the effect was measurable only in the case of the L101P mutant with the strongest protein defect (Figure 5A, D).

To intensify the stress and to investigate the susceptibility of cells with *ABCA3 *mutations to external ER stress-causing agents, transfected cells were exposed to 10 μg/ml of tunicamycin, an inhibitor of N-linked glycosylation (Figure 5B, E). The outcome of such double internal genetic and external tunicamycin pressure on XPB1 splicing was examined. Tunicamycin treatment strongly induced XBP1 splicing with a comparable effect in A549 and WT cells (Figure 5E). However, after exposure to tunicamycin XBP1 splicing was considerably more pronounced in R280C and L101P mutations if compared to A549 cells with WT and R43L mutations (Figure 5B, E). Obviously, although XBP1 splicing was measurable only for the strongest L101P defect under non-stimulated condition, cells with L101P and R280C mutations were significantly more prone to further elevation of ER stress upon exposure to an external stressor.

### L101P and to a lesser extent R280C mutation induce apoptosis of A549 cells

**Figure 6:**
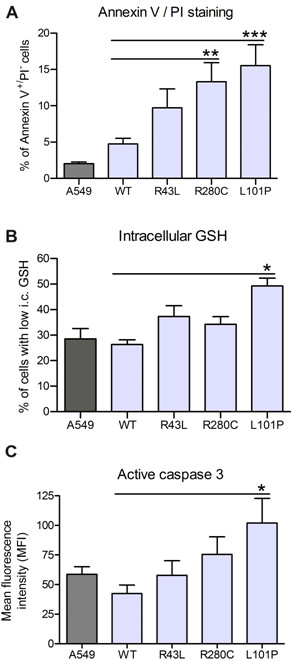
**Apoptosis in A549 cells expressing L101P and R280 mutations**. A549 cells were transfected with pEYFP-N1/ABCA3 plasmids and apoptotic markers were analysed by FACS in the transfected (YFP^+^) population of cells and A549 cells by assaying (A) annexin V^+^/propidium iodide (PI)^- ^staining, (B) intracellular (i.c.) glutathione (GSH) level through coupling of monochlorobimane with GSH, to determine early apoptosis, and (C) intracellular active caspase 3 level (MFI), to detect late apoptosis. Elevated early and late apoptotic markers were detectable in cells expressing L101P mutation, and one early marker (Annexin V) in cells expressing R280C mutation. Results were calculated from six independent experiments; * p < 0.05, ** p < 0.01, *** p < 0.001.

Since prolonged ER stress can activate apoptosis, we analyzed if *ABCA3 *mutations can induce early and late apoptotic markers in A549 cells. In all experiments YFP fluorescence was utilized to determine the population of transfected cells (YFP^+^) and to measure the apoptotic markers exclusively in those cells (Figure 6A, B and 6C).

#### A) Annexin V/PI staining

One of the early signs of apoptosis is translocation of phosphatidylserine (PS) from the inner to the outer leaflet of the plasma membrane. Annexin V specifically binds to the surface-exposed PS of apoptotic cells. Flow cytometry assay of Cy5-coupled Annexin V surface binding showed an increase in the number of annexin V^+^/PI^- ^cells in transfected YFP^+ ^cells in the case of R280C and L101P mutations when compared to WT and R43L indicating an early apoptotic state of those cells (Figure 6A).

#### B) GSH decrease

Glutathione is an essential low-molecular-weight thiol and a major component of the cell antioxidant system [34]. Loss of intracellular GSH is an early hallmark of apoptosis progression and a part of early changes for generation of a permissive environment for the activation of apoptotic enzymes [35]. Intracellular GSH level, measured by flow cytometry of monochlorobimane binding to GSH and generation of a fluorescent adduct, was decreased in the YFP^+ ^cells with L101P protein compared to YFP^+ ^WT, R43L and also compared to R280C (Figure 6B).

#### C) Caspase 3 activation

Activation of caspase 3 is a late apoptotic marker. Caspases are a family of proteins present in the cell as inactive precursors and activated through a cascade of regulated proteolytic cleavages leading to controlled cell death [36]. Caspase 3 is one of the executioner caspases activated at the end of the cascade. Via flow cytometry assay of intracellular active caspase 3 we found an increase in caspase 3 activation in cells expressing ER retained L101P mutant in comparison to the WT and R43L cells (Figure 6C).

In summary, while R43L mutation did not raise apoptotic signaling above the A549 or WT level, R280C mutation increased one early apoptotic marker and ER-localized L101P mutations significantly elevated early and late apoptotic markers, indicating injury of the cells with L101P protein.

### Prolonged ER stress leads to apoptosis through caspase 4 activation in cells expressing R280C and L101P mutations

**Figure 7:**
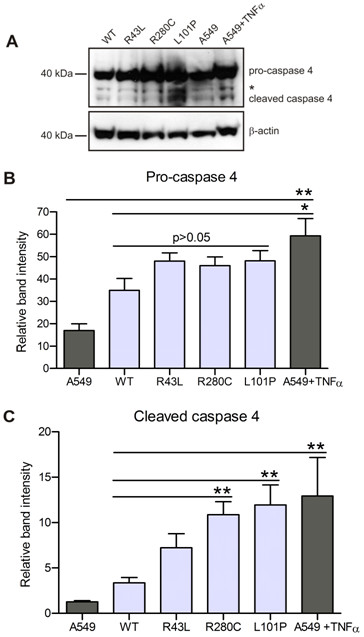
**Apoptotic signaling in cells with L101P and R280C mutations is activated by ER stress**. (A) Immunoblotting on whole cell lysates from A549 cells transfected with pEYFP-N1/ABCA3 and densitometric analyses of (B) pro-caspase 4 and (C) cleaved caspase 4 demonstrated increased caspase 4 cleavage in cells expressing L101P and R280C mutations, as well as after TNFα treatment of A549 (25 ng/ml, 16 h) in comparison to WT and untreated A549. No significant changes in the pro-caspase 4 level in transfected cells with WT, R43L, R280C and L101P mutations were detected but pro-caspase 4 was slightly increased in transfected cells compared to A549 (B). * - unknown band. Six independent experiments were used for densitometric evaluation; * p < 0.05, ** p < 0.01.

To examine if initiation of apoptosis in cells with *ABCA3 *mutations is indeed a consequence of the ER stress signaling, we assessed activation of caspase 4, which is activated by apoptotic stimuli that cause ER stress, but not other apoptotic stimuli [28,37]. Cleavage of caspase 4 leads to proteolytic activation of executioner caspase 3 and to cell elimination. Therefore, activation of caspase 4 identifies ER stress as an apoptosis trigger. Treatment of A549 cells with apoptosis inducing cytokine TNFα resulted in the cleavage of caspase 4 (Figure 7A, B and 7C). Immunoblotting of whole cell lysates from the cells expressing WT or R43L, R280C or L101P mutations showed insignificant changes in pro-caspase 4 level between WT and mutations, but the level of pro-caspase 4 was somewhat higher in transfected cells then in A549 (Figure 7B). In contrast, cleaved caspase 4 in R280C and L101P mutants increased significantly in comparison to WT. Changes measured between R43L and WT were not significant (Figure 7C). This shows that caspase 4 is involved in apoptotic signaling in cells with L101P and R280C mutations and that activation of the apoptotic pathway can be a consequence of the ER stress caused by complete or partial ER retention of the ABCA3 protein.

## Discussion

*ABCA3 *mutations cause surfactant deficiency and fatal respiratory distress syndrome in full-term neonates [10] and chronic ILD of children [12,16,17]. The cellular pathomechanisms of ABCA3-related chronic ILD are probably complex including influence of *ABCA3 *mutations on surfactant homeostasis but possibly also on the fitness and function of AECII as cells for surfactant production, lung repair and immunological defense [38].

In this study we investigated the influence of three *ABCA3 *mutations, R43L, R280C and L101P, on intracellular stress and induction of apoptosis in cultured lung epithelial A549 cells. All three mutations were found in children with ABCA3-associated lung disease being either fatal neonatal respiratory distress syndrome (L101P and R43L [10,14]) or chronic ILD (R280C; own unpublished data, [19]). While cell biology of R43L and R280C mutations was studied here for the first time, L101P mutation was used as a known example of the trafficking/folding defect leading to the ER retention of ABCA3 with no information on ER stress [6,20].

Initial characterization in A549 cells demonstrated that each of the mutations affected the ABCA3 transporter in a different way. We showed correct localization of WT and R43L proteins in LAMP3^+ ^vesicles and dual localization of R280C protein in LAMP3^+ ^vesicles and calnexin^+ ^ER compartment (Figure 1A, B and 1C), indicating less efficient but not abolished R280C trafficking. Similar dual localization is known in the case of other ABCA3 mutants as G122S [6]. R43L and R280C proteins showed WT-processing with two protein bands (Figure 2A) and presence of complex oligosaccharides (Figure 2B) confirming their ability to proceed from the ER to the Golgi. L101P protein remained in the ER, having therefore no complex sugars, and no smaller 180 kDa protein form (Figure 1B and 2A, B) [6,20].

While ER retention of L101P excludes ABCA3 function, the function of R43L and R280C transporters was studied additionally. Since ABCA3 is involved in lamellar body biogenesis [7], in A549 cells, which normally have a low number of compact LAMP3^+ ^vesicles, expression of ABCA3-WT induced biogenesis of LAMP3^+ ^vesicles by increasing their number and size (Figure 3D) [9]. In addition, ABCA3-WT signal was observed as ring-like structures consistent with the ABCA3 presence in the limiting membrane of lamellar bodies (Figure 1 and 3) [5]. In contrast to the WT, expression of *ABCA3 *mutations, especially L101P, impaired biogenesis of LAMP3^+ ^vesicles by reducing their number and size (Figure 3D). This can be a consequence of the inability of these mutated transporters to effectively load lipids into the nascent lamellar bodies.

The uptake assay of NBD-labeled phospholipids PC and PE into ABCA3-HA-positive vesicles demonstrated frequent overlap of the NBD signal, both PC and PE coupled, with the ring-like ABCA3-WT fluorescence and its accumulation in the inner space of the WT-ABCA3 vesicles (Figure 3A, B and 3C). In the case of R43L and R280C mutations such colocalization was rarely observed suggesting functional impairment of R43L and R280C proteins. Similar uptake experiments have been previously described [6]. In contrast to those data, which show diffuse distribution of NBD-PC and NBD-PE fluorescence throughout the cytoplasm, we detected numerous distinct NBD-positive vesicles (Figure 3A, B). The uptake and number of NBD-vesicles observed in the cytoplasm was similar for both phospholipids in A549 cells and in transfected A549, and was also independent of the *ABCA3 *mutation, including L101P. This confirms that for the liposome/lipid uptake through the plasma membrane into the cytoplasm ABCA3 function is dispensable and ABCA3 is solely an intracellular vesicular transporter. Furthermore, we observed an impact of *ABCA3 *mutations on the uptake of both PC and PE. This parallels data published in Cheong *et al. *(2007) [7] which demonstrated decreased incorporation of radiolabeled PC and PE into the lamellar bodies and surfactant of the ABCA3 heterozygote mouse, consistent with a role of ABCA3 in the transport of both phospholipid species. However, this is also in contrast to Cheong *et al. *(2006) [6] showing no uptake of NBD-PE into the lysosome-like vesicles in A549 cells. Therefore, we must point out that it is questionable if the PE transport through functional ABCA3 actually happens *in vivo *or only in an *in vitro *system after exposing cultured A549 cells to NBD lipids.

Continuous overload of the ER compartment with misfolded proteins is harmful for the cell and can trigger apoptotic cell death [22]. L101P protein which accumulates in the ER, caused significant increase of the ER stress and early and late apoptosis markers in the A549 cells (Figure 4, 5 and 6). The ER stress caused by R280C mutation was slightly lower and surface staining with annexin V, as an early apoptosis sign, was the only apoptotic marker detected in R280C cells. This might be a consequence of a dual nature of this protein which could be less harmful for the cell than complete ER retention of ABCA3 and/or possible dependency of the magnitude of its intracellular effects on small unmanageable inconsistencies of an experimental system. The connection between ER stress and apoptosis induction was established through the upregulation of caspase 4 in the case of both L101P and R280C mutations (Figure 7). Correctly localized R43L mutation, despite its influence on the ABCA3 function and lamellar body biogenesis, had almost no impact on stress and apoptosis under any conditions above the range of the WT values (Figure 4, 5, 6 and 7).

ER stress-dependent signaling, examined via XBP1 splicing, was enhanced by exposure of transfected cells to tunicamycin, an ER stressor (Figure 5B, E). In this way the stress pressure imposed on the cells was doubled: 1) genetic background of the *ABCA3 *mutations and 2) exposure to the stress-causing agent. The cells with mutations R280C and L101P, which impair ABCA3 trafficking, were more prone to further XBP1 splicing than the WT or A549. This is interesting if known that viral infections (e.g. RSV, herpes virus) or cigarette smoke are common outside factors which, as tunicamycin, elevate ER stress [39,40]. Doan *et al. *(2007) [12] observed onset of ABCA3-associated ILD in children following exposure to cigarette smoke and Young *et al. *(2008) [16] described a teenage patient with a late onset of ABCA3-related disease with fibrosis following the beginning of cigarette consumption. If *ABCA3 *mutations can raise susceptibility of AECII to external stress, additional exposure to outside stressors as respiratory viral infections or smoke might contribute to or even trigger genetic ILD.

Misfolding and ER retention of other lipid ABC transporters of the A subfamily, as ABCA1 and ABCA4, cause Tangier disease and Stargardt macular dystrophy, respectively [41,42], but their influence on ER stress and apoptosis is unknown. The most common mutation ∆F508 of another ABC transporter of the C subfamily, CFTR/ABCC7, leading to cystic fibrosis, results in CFTR misfolding and retention in the ER, and can raise stress and activate UPR [43]. The concept that ER stress and apoptosis lead to lung disease has been explored recently when it was demonstrated that ER stress and apoptosis of AECII are involved in the injury of lung epithelium in idiopathic pulmonary fibrosis and SP-C deficiency [25,26]. Also, expression of SP-C mutations resulting in proSP-C misfolding and aggregation increases ER stress, activates UPR and induces apoptosis in A549 or HEK293 cells [27,28]. SP-C and ABCA3 are both AECII-expressed proteins essential for surfactant homeostasis and both lead to genetic ILD equally variable in the age of onset, severity and pathology [44]. Therefore, it is interesting to see that the common mechanisms underlying both types of genetic ILD must exist and our data show that they probably encompass the ER stress and apoptosis of AECII.

## Conclusion

Apparently, the effect of *ABCA3 *mutations on homeostasis of lung epithelial cells depends on the type of the ABCA3 protein defect. More work is necessary to verify if ER stress and apoptosis of AECII are indeed present in (fibrotic) lung tissue of patients with *ABCA3 *mutations. This might help to understand or even predict the disease course, or consider new therapy options (as protein rescue with chaperones) depending on the type of the ABCA3 protein defect. In conclusion, we have presented the cell biology study of two new *ABCA3 *mutations and demonstrated that those mutations which influence partially or completely ABCA3 trafficking raise intracellular stress and susceptibility to it, and induce apoptotic cell death signaling. Similar as proposed for SP-C deficiency, intracellular stress and apoptosis of AECII might play a role in pathogenesis of ABCA3-related lung disease.

## List of abbreviations

ABC transporter: ATP binding cassette transporter; AECII: alveolar epithelial type-II cells; ILD: interstitial lung disease; UPR: unfolded protein response; ER: endoplasmic reticulum; YFP/GFP: yellow/green fluorescent protein; HA: hemagglutinin; IPF: idiopathic pulmonary fibrosis; SP-C: surfactant protein C; BiP: binding immunoglobulin protein; RT-PCR: reverse-transcription PCR; XBP1: X-box binding protein 1; TM: tunicamycin; FACS: fluorescence-activated cell sorting; NBD: 7-nitrobenz-2-oxa-1,3-diazole; PC: phosphatidylcholine; PE: phosphatidylethanolamine; LAMP3: lysosomal-associated membrane protein 3; GSH: glutathione; TNFα: tumor necrosis factor α CFTR: cystic fibrosis conductance regulator.

## Competing interests

The authors declare that they have no competing interests.

## Authors' contributions

SK, NW, MG and AHec conceived and designed the study. NW, SK, EK, AHec, AS and MW performed the experiments and analyzed the data, which were interpreted by SK, AHec, MG and NW. AHolz contributed the pEYFP/ABCA3-WT plasmid. SK wrote the manuscript which was edited by MG, NW, EK and AHec. All authors have read and approved the final manuscript.

## Authors' information

Until 2009 Sunčana Kern (SK) published as Sunčana Moslavac. The present study will form part of the MD thesis of Nina Weichert.
